# Language neuroscience in the operating room: neurosurgical considerations for multilingual brain tumor patients

**DOI:** 10.3389/fonc.2025.1648154

**Published:** 2025-11-04

**Authors:** Sebastian Sanchez, Matthew Tate

**Affiliations:** Department of Neurological Surgery, Northwestern University, Chicago, IL, United States

**Keywords:** multilingual brain mapping, awake craniotomy, multilingualism, neurolinguistics, brain tumor

## Abstract

Understanding the neural basis of language is critical for neurosurgical procedures involving awake brain mapping. Advances in neuroimaging have helped reshape traditional models of language organization, highlighting dynamic, bilateral cortical-subcortical hodotopical networks that support language processing through a ventral semantic-focused stream, and a dorsal phonological-focused stream. In the operating room, especially during awake craniotomies for glioma resection, this nuanced understanding of human language is key for minimizing deficits and optimizing outcomes, with additional considerations for bi- and multilingual patients. Direct Electrocortical Stimulation (DES) remains the clinical the gold standard for intraoperative mapping, often supplemented with electrocorticography (ECoG) and pre-operative functional magnetic resonance imaging (fMRI). Selecting appropriate language tasks and assessing linguistic proficiency across all languages involved are crucial for tailoring individualized mapping strategies. A detailed linguistic profile, considering factors such as language proficiency, use, and age of acquisition, may help anticipate functional reorganization patterns and surgical planning. This review synthesizes current neuroscientific literature and insights into language and multilingualism, explores the effects of brain pathology on language processing, and outlines clinical best practices for language mapping in multilingual patients undergoing awake neurosurgery.

## 1 Introduction

The functional organization of language in the brain has captivated scientists and the public alike for nearly two centuries. For most of the field’s existence, classical models of language representation have largely focused on simplified notions of neurological language representation, mostly limited to the familiar “Broca and Wernicke” areas. However, the advent of modern neuroimaging and direct mapping techniques has revealed the degree to which the classical models are underspecified and inadequate in explaining the complex neurological substrate for the cognitive phenomenon that is human language (1). It is now widely recognized that human language relies on the orchestrated cooperation of various intricate bilateral, cortical and subcortical networks.

For the neurosurgeon tasked with resection of a tumor in a brain area implicated in language, understanding the behavior of language networks is paramount for the preservation and maximal retention of cognitive ability. The extent to which specific regions of neurological tissue are involved in language networks is tested intraoperatively during awake craniotomies using direct electrocortical stimulation (DES). The widespread use of DES in awake neurosurgeries to preserve patients’ cognitive abilities such as language and even musical performance has not only drastically improved the patient quality of life following surgery but also the scientific understanding of language cortical representation. As the most direct mechanism of assessing neurological activity, DES is widely recognized as the gold-standard in measuring brain function.

With a growing majority of the human population being bilingual or multilingual, understanding the basis for the coexistence of two or more language systems in the brain is of increasing relevance. In the context of surgery, the multilingual brain has unique functional characteristics that must be accounted for in order to achieve maximal retention of linguistic abilities for each of a patient’s languages.

## 2 Neuroscientific principles of language and multilingualism

Prevailing theories for human language organization share an emphasis on dynamically modulated networks that integrate bilateral, cortical and subcortical networks. One salient example is Hickok and Poeppel’s (2) model for the functional organization of speech processing in the human brain which proposed a dual-stream model for language consisting of two broad distinct but interacting pathways. This model, analogous to the ventral-dorsal stream model for visual processing (“what” and “how” pathways originating in the occipital lobe, respectively), highlights a corresponding functional basis for auditory speech processing (3). While the original proposal of this model focused largely on the cortical foundations of the respective streams, data from DES studies on cortical and subcortical regions helped attribute the role of specific white matter tracts to this theoretical framework (4).

The dual-stream model has evolved and adapted over the last two decades with contributions from functional imaging, tractography, and DES data (3). The first of these parallel interconnected pathways is a ventral stream for lexical-semantic processing of speech signals that involves the superior and middle temporal lobes bilaterally, connecting to the ventrolateral prefrontal cortex via the extreme capsule, uncinate fasciculus (UF), and inferior fronto-occipital fasciculus (IFOF; 1, 4–6). The other is a dorsal stream that involves the posterior dorsal-most aspects of the temporal lobe and parietal operculum, and the posterior frontal lobe via the superior longitudinal fasciculus (SLF)/arcuate fasciculus (AF) system that includes the white matter subcomponents AF, SLF I, II, III and SLF-tp (6). This pathway is mostly involved in the translation of speech signals to articulatory representations supporting speech production and phonological working memory (1, 7). Additionally, the dorsal stream likely plays a role in sensorimotor integration with the ventral premotor area and inferior frontal gyrus (IFG) pars opercularis through the mapping of phonological information onto articulatory motor representations (3, 6).

Similar to the Hickok-Poeppel model, the Rauschecker-Scott model has built upon this foundation with evidence from non-human primates. These authors argue that the dual-stream model is hierarchically and topographically organized in a way that is continuous with our evolutionary development of auditory processing mechanisms (8). Additionally, they argue that the sylvian parietotemporal region (involved in sensorimotor integration characteristic of dorsal stream) is also responsible for auditory spatial processing. With evidence from anatomic and physiologic studies of primate auditory cortex along with human DTI data, their model proposes a loop originating from the primary auditory cortex with one branch extending postero-dorsally towards premotor areas, and another branch extending antero-ventrally towards the inferior frontal gyrus. Thus, Rauschecker & Scott (8) extend the dual-stream scheme to close the loop between speech perception and production.

Overall, this dual-stream framework recognizes the bilateral nature of language while still maintaining a level of left-hemisphere dominance. While this framework developed with the invaluable contribution of modern neuroimaging data, it is worth noting that this schema for language processing in the human brain has its origins in classical models. Chang et al. (3) astutely point out in their review of modern neurolinguistic theory:

The dual stream model of language processing has nonetheless had a dramatic influence on contemporary thinking about localization, and many language studies are now interpreted in this framework. It should be pointed out, however, that these general concepts were originally conceived by Wernicke in 1874. At that time, he already proposed that sensory representations of speech in the posterior temporal lobe interfaced with two distinct systems, a broadly distributed conceptual system for comprehension and the motor system to help support the production of speech. Therefore, the major contribution from recent models has been the refinement of anatomical localization, specification of language subprocesses, and most importantly, confirmation using best available evidence from the past half decade with modern imaging and careful lesion-deficit studies.

Thus, while the dual-stream model represents a major innovative advancement beyond the Wernicke’s conceptual framework, formalizing a bilateral evidence-based organization of language networks, it also provides a bridge between classical and modern perspectives. Contemporary frameworks developed in the last decade with evidence from DES studies and connectomics, however, have extended this framework towards a “hodotopic” understanding of language networks (9). This view (from the Greek *hodos*, “path” and *topos*, “place”) is a paradigmatic shift towards a highly distributed and dynamic understanding of the central nervous system which holds that complex cognitive phenomena such as language emerge from dynamic, plastic, and highly interconnected cortical-subcortical pathways rather than discrete cortical loci (9, 10) In multilinguals, hodotopy accounts for the idiosyncratic and partially overlapping neural representations of different languages, as well as differing reorganization patterns shaped by individual language history and the specific tracts affected by pathology (11). This perspective provides a step forward in explaining the interaction of various languages in a multilingual subject, but it is not wholly sufficient per se. When it comes to understanding the maintenance of multiple languages in the brain, many critical factors must be considered, namely age of acquisition (AoA), proficiency level, and cognitive control mechanisms, all of which impact the inter-subject variability of language networks.

With regards to a model for multilingualism, the literature suggests shows that all languages within a subject are largely underpinned by shared networks, but with critical nuances. For example, on the basis of phonology, multilinguals may have additional processing demands as a product of competing representations extending from articulatory planning to post-articulatory monitoring. On grammar, the sharing of biological substrates across languages exists with variations in activation patterns that depend on aforementioned factors such proficiency, language distance, and AoA (12). Low proficiency level and/or exposure in multilinguals may impact the biology of lexico-semantic processing by requiring greater recruitment of the prefrontal cortex (12).

Multilingual language control relies on executive attention networks in addition to language networks, implying that multilingualism has neurological effects beyond language processing with potentially beneficial effects at the neural and cognitive level (12). For example, lifelong bilingualism has been shown to be associated with greater white matter integrity, enhanced cognitive reserve in later life, and structural differences in regions involved in language control and executive function (13). Furthermore, studies on multilingualism indicate that multiple languages in the brain requires the co-activation of dynamic neural mechanisms for language switching and inhibition. One such cognitive linguistics experiment by Starreveld et al. (14) demonstrated the co-activation effect of the non-target language during word production in English-Dutch bilinguals. This co-activation and constant need for monitoring of language control in multilingual individuals is believed to contribute to the enhancement of neuroplasticity (15).

Despite the general consensus in the literature regarding overlapping biological substrates for distinct languages, this theoretical schema is not always observed in clinical practice, such as in neurosurgical contexts where language mapping is required. A recent compelling systematic review by Połczyńska' & Bookheimer (16) on awake brain mapping studies in bilinguals analyzed 28 studies with 207 patients and found evidence for generally *separate* cortical areas for different languages in both anterior and posterior sites. In cases in which there was overlap between L1 (first language) and L2 (second language), the relationship was explained by either early L2 AoA and small linguistic distance, a quantification of the phylogenetic relationship and mutual intelligibility between language families. This review suggests that AoA, proficiency, and exposure are associated with increased neuroanatomical overlap. In other words, these data suggest that frequent everyday use of both languages can lead to an increased sharing of neural substrates for different languages. Połczyńska & Bookheimer (16)’s review demonstrates that the heterogeneity of a multilingual’s linguistic profile has demonstrable effects on a patient’s language mapping pattern, which has critical implications for surgical planning that will be further discussed in Section 6 of this review. In sum, these studies highlight the high degree of individual variability in language neuroanatomical overlap, influenced by diverse factors such as cognitive control, linguistic similarity, a patient’s unique language usage behaviors (16–18).

## 3 Effects of brain pathology on language networks

Instances of pathology can be particularly insightful in understanding the neurobiology of language in multilingual patients as they reveal how language networks adapt, reorganize, and fail under unique pathological conditions. This understanding is vital for clinical care and research given that patients with glioma have considered language to be the most important function to preserve, even over motor ability, memory, and problem solving, (19). Important pathological factors influencing language outcomes include the type and location of the lesion, as well as, in the case of brain tumors, the degree of white matter involvement and the rate of tumor growth.

- Pathology type: different neurological insults exert substantially different influences on language networks. Stroke, for example, characteristically produces abrupt, focal disruptions with well-defined acute, subacute and chronic recovery phases. Gliomas (especially low grade gliomas), however, induce progressive, network-level remodeling that begins preoperatively and continues after resection. (20). This pattern of remodeling may lead to transient aphasia but better long-term recovery as a result of controlled function-preserving resections (20).
- Lesion location is also a key determiner for linguistic deficit type. Gobbo et al. (21) found that temporal lobe tumors were more often associated with co-hyponym errors, whereas frontal tumors produced synonym emissions in tasks involving hierarchical lexical retrieval. These findings highlight that distinct cortical regions may differentially contribute to semantic categorization and lexical control, which may be especially complex in multilinguals.
- White matter tract involvement can also significantly alter language function. Infiltrative tumors such as gliomas are known to spread along white matter tracts and blood vessels to cause demyelination and vasogenic edema (19). Disruption of language tracts, such as the arcuate fasciculus (AF), inferior fronto-occipital fasciculus (IFOF), and superior longitudinal fasciculus (SLF), can substantially impact inter- and intrahemispheric communication that promotes compensatory recruitment of homologous or perilesional areas in certain cases (19, 22). Involvement of language white matter tracts such as the IFOF is also associated worse prognosis and permanent language impairments and unique language reorganization patterns in bi-/multilinguals (19, 23, 24).
- Tumor growth rate is another important factor in language outcomes as slower growing tumors, such a slow grade gliomas, can trigger language network reorganization via neuroplasticity mechanisms that allow transfer of linguistic faculties from infiltrated areas to structurally and functionally preserved regions (25). Evidence from resting state electroencephalography (rs-EEG) supports this view of dynamic adaptation, with one study finding that low grade glioma patients exhibited increased delta and theta activity pre- and post-operatively with preoperative theta power (26). Notably, meningioma patients did not exhibit increased slow wave activity compared to healthy controls but also suffered similar post-operative language impairments, suggesting distinct electrophysiological mechanisms of dysfunction between infiltrative and compressive pathologies.

Brain pathology may impact monolinguals differently than bilingual and multilinguals. ReFaey et al. (27) investigated bilingualism as a prognostic factor in a retrospective review of 56 patients (14 bilingual) undergoing left-sided awake craniotomy. Bilingual patients demonstrated higher tolerance to direct electrical stimulation (DES) currents and fewer intraoperative seizures (although not statistically significant). These findings suggest that bilingualism may enhance patients’ ability to engage distributed and redundant neural representations that buffer against surgical or pathological disruption. Furthermore, functional imaging studies in bilinguals show that L1 and L2 have both shared and distinct subcortical connectivity patterns, and tumor-induced damage may result in differential deficits and recovery trajectories for each language (25). A further discussion on the impact of brain pathology in multilinguals, and specifically how tumors may affect cortical and subcortical reorganization in this population, is provided in Section 5.

## 4 The use of direct electrocortical stimulation for the study of language networks

Awake brain mapping through direct electrocortical stimulation (DES) is unapparelled in many respects for allowing resection of tumors in formerly inaccessible areas (such as the insula), its protection against permanent post-operative language impairment, and its direct insight into language representation (19). DES provides several advantages over noninvasive imaging. For example, it is causal – directly disrupting neural activity with millisecond-level temporal precision – rather than correlational, such as fMRI which relies on hemodynamic responses that may be subject to neurovascular uncoupling in the context of an insult (15). Intraoperative DES studies have shed light on the variability of language representation across subjects. One of the early landmark DES studies investigating essential language sites across 117 patients demonstrated a definitive mosaic of cortical representation and the need for revision of the classical models (28).

More recently, intraoperative DES studies have revealed more specific characteristics of language networks, de-emphasizing strict interpretations of the classical model in favor of bilateral probabilistic maps for anatomic epicenters for language functions, such as phonologic and semantic hubs, subserved by parallel networks, which is more practical for consideration in neurosurgical settings (9, 29, 30). Mugler et al. (31) found that articulatory gestures and phonemes are differentially represented in the precentral and inferior frontal gyri, highlighting the role of the primary and premotor cortices. This provides evidence for the importance of the sensorimotor system in speech production and the embodied nature of cognitive phenomena such as language (32). Hsieh et al. (33) analyzed cortical sites involved in speech arrest and language errors, finding that these regions were more strongly associated with inter-community connectivity (module connectors), which suggests that cortical sites critical for language function serve as key connectors between distinct language subnetworks, facilitating communication and integration across the broader language network.

The accuracy of language mapping has improved over recent decades, especially when combined with preoperative techniques such as resting-state fMRI (rs-fMRI), MRI-based tractography, and navigated transcranial magnetic stimulation (nTMS), which boasts a high sensitivity and good correlation with intraoperative DES (34, 35). In the context of multilingualism, the use of electrocorticography (ECoG) in multilingual epilepsy patients in conjunction with DES was able to identify language-specific sites that DES could not in 75% of patients, providing evidence that ECoG can complement DES in discriminating the cortical representations of separate languages (36).

It is worthy to note the limitations of DES, as it can only be accomplished during neurosurgery and can therefore only be used to investigate neurological function in the setting of pathology, such as a tumor. This makes it challenging to generalize the findings to healthy function and normative models. Prior to noninvasive neuroimaging techniques such as fMRI and EEG, brain function could only be investigated in the context of pathology – e.g. a lesion in a particular region identified postmortem may have been linked to a particular cognitive deficit while the patient was alive. This gave rise to the misleading impression that broad functions such as language, emotions, and memory could be entirely localized. Now, it is understood that cognitive phenomena can rarely be linked to just one site. This mode of reasoning, known as the lesion-deficit tradition, dominated the scientific understanding of the brain until the late 20^th^ century and is important to keep in mind when interpreting the conclusions drawn from DES studies on patients who obligatorily will have a neuropathology (15).

## 5 Language reorganization in multilingual tumor patients

Many aspects of the neuroscience of multilingualism remain debated, and the reorganization of multiple language networks in the setting of pathology has even more research potential. While it is known that tumor growth can induce language reorganization in monolinguals, there is not as much known for bilinguals or multilinguals. In a study of five bilingual tumor patients who underwent awake craniotomy, Quiñones et al. (25) found that brain tumors lead to reorganization of language networks to the right hemisphere and ipsilesional left hemisphere areas, and that L1 and L2 followed distinct reshaping patterns following surgery. The authors conclude that neuroplasticity impacts the compensatory involvement of executive control regions, “supporting the allocation of cognitive resources as a consequence of increased attentional demands” (25). Thus, bilingual brains likely follow different reshaping patterns after tumor resection.

A systematic review of 7 studies with 25 multilingual patients with left frontal lobe tumors (mostly gliomas) who underwent language mapping indicated heterogeneity in the level of overlap of cortical sites subserved by L1, L2, and L3, finding that L3 tends to be more unpredictable (18). In general, L1 and L2 shared many sites near the pars triangularis and opercularis (18). The review agreed with the findings from Połczyńska & Bookheimer (16) in that languages learned earlier tend to have a higher degree of shared cortical sites than those learned later, and that languages with a later AoA generally exhibit activation in a greater number of sites, especially distal ones (37). These data support the notion that younger AoA (and likely higher proficiency and exposure level) are correlated with more cortical integration of different language networks.

However, these findings are not always the case. For example, one study of 13 multilingual individuals with lesions in the left, dominant hemisphere found the opposite trend where younger AoA was associated with greater, more distinct cortical representation than later acquired languages (38). Moreover, this study found that late-acquired languages largely overlap with early-acquired language sites, with the highest overlap (71%) occurring between early- and late-learned languages (38). Notably, the patients in this cohort were a mix of fast-growing tumors (n=5) and slow-growing tumors (n=7). As discussed in Section 3, tumors may trigger reorganization to the cortex adjacent to the lesion or within the same hemisphere in a network of areas that are not language typical (17). Importantly, the temporal pattern of lesional growth is known to cause differences in language function redistribution, with slow-growing lesions, such as low-grade gliomas, allowing for more effective neuroplastic reorganization that may lead to better outcomes (22). This difference in behavior of slow and fast-growing lesions may explain the results found by Fernández-Coello (38). The heterogeneity in the literature highlights the complex, multifaceted contribution of variables related to tumor pathology as well as patient-specific language profile in characterizing their overall effect on patient outcomes. The final section of this review proposes a strategy to standardize data collection and while acknowledging logistical challenges in patient care to address the heterogeneity in the field.

## 6 Discussion

### 6.1 Clinical and surgical implications: current methods and challenges for assessing language in brain tumor patients

Current protocols for language testing in the perioperative setting for a patient scheduled to undergo awake craniotomy requires substantial teamwork and interaction from a diverse and multidisciplinary healthcare team, including neurosurgeons, neuropsychologists, neurophysiologists, anesthesiologists, etc. One review of 178 studies on indications for awake surgery for glioma resection found that in 84% of them, monitoring of language spectrum functions was the main indication for awake craniotomy (39). This article also found that the most common documented exclusion criteria for awake craniotomy included inability to cooperate from psychological conditions, severe language deficits, and existing medical conditions; age and tumor histology were not standardized variables for exclusion (39). Generally, awake craniotomy is recommended for glioma patients in which testing language, sensorimotor, or visuospatial functions is relevant given that they do not meet any of the mentioned exclusion criteria. In select patients, preoperative imaging may include functional MRI (fMRI) with language tasks to identify critical language nodes. While this approach is not standardized across all languages, it is occasionally incorporated into preoperative planning. While fMRI is valuable for preoperative planning, it cannot yet replace intraoperative mapping as language protocol activations show only a 29–52% positive predictive value for nodes identified through DES (3). fMRI also tends to underestimate critical articulatory regions outside the inferior frontal gyrus, and while activations are highly sensitive, they lack specificity because critical nodes cannot be reliably distinguished from less essential ones (3). In addition to fMRI, diffusion tensor imaging (DTI) sequences are sometimes acquired to evaluate white matter tract involvement; these studies are typically covered by insurance and add minimal burden if an fMRI is already being performed.

Neuropsychological assessment is routinely conducted, including cognitive testing and intraoperative surgical task training, facilitated by neuropsychologists; however, indications and perioperative procedures for awake craniotomy patients are not fully standardized (39). At Northwestern Memorial Hospital, translators are occasionally used for multilingual patients, and intraoperative language mapping is generally performed in one language. When intraoperative testing in multiple languages is required, most often for patients who do not speak English, video interpreter services may be employed to assist with intraoperative tasks such as counting, naming, and object identification. However, such testing is limited by time constraints, resource availability, and demand for specialized personnel to administer detailed psycholinguistic evaluations. Accounting for various languages adds additional layers of complexity to an already complex surgical procedure. It is not standard practice to adjust the protocol for bilingual and multilingual patients who require individualized language assessment since there is little perioperative linguistic data to support such alteration of procedures. However, our review argues for systematically assessing language ability in multilingual patients undergoing awake brain mapping as an essential step for successful preservation of function and post-operative quality of life. In addition to the clinical benefit, it is also good practice for research since perioperative language data is necessary for assessing multilingual language organization in the brain (40). Unfortunately, the current field is generally limited by a lack of standardization (41, 42).

Accurate perioperative language assessment is necessary for detecting patients’ deficits and language-specific cortical regions (41). De Martino et al. (41) systematically analyzed literature involving brain tumor patients from 1991-2021, finding a need for individualized, tailored approaches for multilingual assessment while recognizing that this may lead to inconsistency across neurosurgical teams. Generally, the review found a great heterogeneity in the procedures used to measure dimensions that impact language organization (age and type of acquisition, exposure, proficiency, and use) and the preoperative language assessment of all languages spoken by a patient. The review did find however that the intraoperative task used during language mapping, the picture naming task, is highly common. The authors state:

Noteworthy, no strong statement was reported about whether and to what extent AoA and proficiency scores helped planning intraoperative procedures (e.g., selecting languages, tasks, stimuli, and stimulation sites) nor if they had an impact on the outcome of surgery. This finding alone shows that information on AoA and proficiency has not been properly used to shed light on the cerebral organization of multiple languages. Such a bias could be neutralized if multilingual patients eligible for awake surgery were systematically questioned to obtain objective measures of their multilingualism.

Lamentably, the authors found that 50% of proficiency scores in the reviewed literature are from self-rating or self-report (41), which is known to be a notoriously poor predictor of true linguistic proficiency (15). Only about 9% of proficiency scores are obtained from formal linguistic assessment (41). Overall, there is a strong need for operationalization of procedures. The following section proposes a practical method for obtaining necessary perioperative linguistic variables in the clinical context.

### 6.2 The linguistic history and multilingual profile

**Figure 1:**
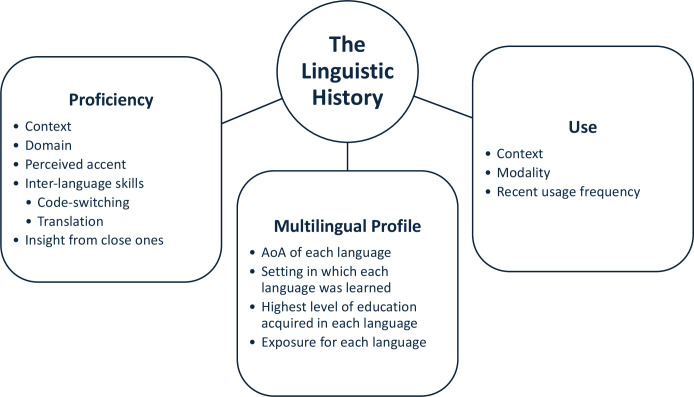
Recommendation of awake brain mapping perioperative linguistic variables as described by De Martino et al. (41). An accurate and comprehensive linguistic history is necessary for multilingual brain tumor patients undergoing awake mapping in order to properly assess functional changes as a result of both tumor-induced and surgically-induced neuroplastic language reorganization.

With multilingualism being far from an all-or-nothing phenomenon, it is necessary to characterize the heterogenous profile of multilingual patients in a way that is sensitive to the distinct and interacting variables that affect the neurobiology of multilingualism (12). De Martino et al. (41) outline best practices recommendations for perioperative language assessment for multilingual patients undergoing awake mapping. They describe three main categories of relevant experience-related linguistic factors. First is the Multilingual Profile, which evaluates AoA, setting in which it was learned, education, and exposure for each language. Second is the Use of each language to assess language context, modality, and recent usage frequency. The last factor is Proficiency, which examines language context, domain, perceived accent, and skills in code-switching, translation, and qualitative information from family/friends on the patients’ impairments. These categories are depicted in **Figure 1**. Intraoperative testing should use object naming, sentence completion, and translational/switching tasks relevant to the patient. It is also recommended that assessments are validated against control groups of healthy subjects with similar linguistic backgrounds.

Based on the need for operationalization and synthesis of the literature gleaned from this review, we recommend the standardization of protocols for the acquisition of perioperative linguistic variables for multilingual patients scheduled to undergo awake craniotomy. The comprehensive linguistic profile should rely on a combination of semi-structured interview and objective tests. The interview should capture core variables known to shape multilingual language organization, including:

- Age of acquisition (AoA) for each language.
- Frequency and context of use across settings such as home, school, work, and travel.
- Highest level of education completed in each language.
- Self-reported proficiency.
- Caregiver-reported insight on patient’s language proficiency and use to account for potential under- or overestimation by the patient.

To complement these subjective measures, objective testing should be performed to gather a validated estimate of patient language proficiency. For research purposes, we recommend administration of abbreviated reading comprehension tasks aligned with aligned with the Common European Framework of Reference for Languages (CEFR). These validated tests are currently available in 15 languages, with comparable alternatives used for languages not represented in the testing bank. During the postoperative period, longitudinal follow-up tracking of recovery patterns and language outcomes over time is advised. This approach offers both immediate clinical value by identifying languages at potential risk, and long-term research value, by providing structured evidence of how multilingualism interacts with brain plasticity and recovery in the neurosurgical setting.

The patient-tailored procedure provided here is our recommendation to clinicians and researchers aiming to collect evidence-based perioperative linguistic data to enhance preoperative and intraoperative language mapping for multilingual patients. Proficiency should be assessed using both subjective and objective ratings across various dimensions, including different contexts, modalities, linguistic domains, perceived accents, spontaneous language switching, cross-linguistic flexibility, translation engagement, effective communication skills, and family/friends and patient perceptions of impairment in different languages. It is crucial to operationalize and treat all these variables comparably in awake surgery settings for reliable findings supported by formal statistical analyses in cross-linguistic studies (41).

The recent development of natural language processing (NLP) tools has the potential to significantly improve the reliability of proficiency scores and perioperative language testing. These new technologies are capable of extracting lexical, syntactic, semantic, and acoustic features from patient language samples, in essence providing digital biomarkers that detect and subtype language disorders often with greater sensitivity and efficiency than traditional neuropsychological tests (43, 44). One systematic review assessing the use of NLP for language testing found a pooled area-under-the-curve estimate exceeding 0.85, noting that these models frequently outperformed traditional assessments while requiring only short speech samples or existing electronic health record text (43). For patients with brain tumors, NLP-based tools can quantify subtle changes in language output that reflect underlying network reorganization and neuroplasticity, supporting longitudinal monitoring and individualized assessment of recovery or decline. In multilingual patients, these tools offer the ability to analyze multiple languages that may follow distinct deficit/recover patterns in a scalable way. One example of this tool is Open Brain AI (OBAI), which utilizes NLP, machine learning (ML), speech-to-text transcription, and statistical and probabilistic models. It currently supports clinical assessment in 14 languages in a variety of neurolinguistic contexts such as aphasia and dementia (45). These types of clinical tools could serve as an efficient means to assess perioperative language assessment. However, data for its use in this clinical population is sparse and clinical adoption will require attention to algorithmic bias, cultural-linguistic representativeness, privacy standards, and explainability (43).

### 6.3 Application and utility of perioperative linguistic data for surgery

The role of perioperative linguistic data in clinical management of multilingual brain tumor patients is undoubtedly important, yet its nature is incompletely characterized due to a relative lack of operationalization and small sample sizes in existing studies. To establish clinically meaningful guidelines, larger studies with standardized protocols are essential. It is well recognized that language factors such as AoA, proficiency, and use frequency have an influence on cortical language representation and should guide neurosurgical approaches. Accordingly, the use of preoperative imaging modalities such as fMRI and DTI can assist in surgical planning, and comprehensive testing of all a patient’s languages, both preoperatively and intraoperatively, is essential to ensure equitable and function-preserving outcomes for bilingual and multilingual individuals.

Implementing a detailed linguistic profile will enable precise categorization of each patient’s multilingual status and support informed assessment of whether their languages may require tailored surgical approaches. By integrating variables from the linguistic history, proficiency scores, and analyses derived from NLP-based technologies, we can infer the likely degree of cortical overlap, symmetry and asymmetry of a patient’s language networks, and whether there is a clinical need to assess additional languages intraoperatively to ensure preservation of L2 function. Although further data are required to fully understand how these variables influence language representation and reorganization in the multilingual brain, systematically capturing them offers meaningful value. At present, this information may be used to guide surgical decision-making while also contributing to a growing body of evidence that will be critical for elucidating these complex effects in a diversity of patients.

### 6.4 Conclusion

The evolving neuroscientific understanding of language as a dynamic, distributed network has profound implications for neurosurgical procedures, particularly in an increasingly bi- and multilingual population. Language representation varies widely across individuals, influenced by linguistic factors such as age of acquisition, proficiency, and language distance. Tumor-induced reorganization further complicates this landscape, emphasizing the need for patient-tailored surgical approaches. While DES remains a powerful intraoperative tool, systematic analyses are hindered due to a lack of standardized perioperative language assessment protocols. Formalized proficiency testing, potentially augmented by advanced computational tools such as NLP, can bridge this gap. Furthermore, the precise influence of a patient’s tumor characteristics and detailed linguistic profile has yet to be formally analyzed as possible predictors of postoperative language outcomes or deficits, and the degree of cortical overlap observed between languages. These open questions highlight the field of neurosurgery’s powerful role in elucidating the nature of complex phenomena that define human cognition.
